# Study partner types and prediction of cognitive performance: implications to preclinical Alzheimer’s trials

**DOI:** 10.1186/s13195-019-0544-6

**Published:** 2019-11-27

**Authors:** Michelle M. Nuño, Daniel L. Gillen, Joshua D. Grill, for the Alzheimer’s Disease Cooperative Study

**Affiliations:** 1 Institute for Memory Impairments and Neurological Disorders, University of California, Irvine, Irvine, CA USA; 2 Department of Statistics, University of California, Irvine, Irvine, CA USA; 3 Department of Neurobiology and Behavior, University of California, Irvine, Irvine, CA USA; 4 Department of Psychiatry and Human Behavior, University of California, Irvine, Irvine, CA USA

**Keywords:** Study partners, Alzheimer’s disease, ADCS-PI

## Abstract

**Background:**

Alzheimer’s disease (AD) clinical trials require enrollment of a participant and a study partner, whose role includes assessing participant cognitive and functional performance. AD trials now investigate early stages of the disease, when participants are not cognitively impaired. This gives rise to the question of whether study partners or participants provide more information in these trials.

**Methods:**

We used data from the AD Cooperative Study Prevention Instrument Project (ADCS-PI) to compare participant and study partner predictions of the participant’s current and future cognitive state. We used the Cognitive Function Instrument (CFI) as a measure of evaluation of the participant’s cognitive status and the modified ADCS Preclinical Alzheimer’s Cognitive Composite (mADCS-PACC) as an objective measure of cognition. Stratifying by cognitive status and study partner type and adjusting for other predictors of the participant’s cognitive state, we used random forests along with estimated mean variable importance (eMVI) to assess how well each member of the dyad can predict cognitive state at current and later visits. We also fit linear regression models at each time point and for each scenario.

**Results:**

Participants were better at predicting future cognitive status compared to their study partners regardless of study partner type, though the difference between participants and partners was greatest for non-spousal dyads in the lowest-performing quartile. Cross-sectional assessments differed substantially by dyad type. Within the lowest cognitive performance quartile, participants having a non-spousal study partner outperformed their partners in assessing cognition at later times. Spousal partners, in contrast, outperformed participants later in the study in predicting current cognitive performance.

**Conclusions:**

These results indicate that participants tend to be better at predicting future cognition compared to their study partners regardless of the study partner type. When assessing current cognition, however, spousal study partners perform better at later time points and non-spousal study partners do not provide as much information regarding participant cognitive state.

## Background

Most Alzheimer’s disease (AD) clinical trials have tested candidate therapies in patients with dementia. Intervention at this stage may be too late, however, due to prevalent neurodegeneration [1]. Therefore, the focus has shifted toward preclinical stages of the disease, before overt clinical symptoms are present. AD trials at every disease stage require the enrollment of a participant and a study partner.

In AD dementia trials, study partners ensure informed consent, assist with protocol compliance, and are the source of information for a variety of trial outcome measures [2]. Not only must a participant have a study partner who is willing and able to enroll in the trial, this study partner should be able to provide high integrity information about the participant’s daily life and cognition [3].

While the rationale for the study partner requirement is clear in AD dementia trials, it is less obvious for preclinical AD trials, in which participants can provide consent and ensure trial compliance. Furthermore, the study partner requirement may be a barrier to recruitment in these trials [4]. It is therefore important to understand the value of the information study partners provide about the participant’s cognitive ability during the trial. Moreover, certain types of study partners in dementia trials may provide data of differing levels of integrity. Previous work has shown that spousal study partners provided more accurate information than other study partner types when considering participants with Alzheimer’s disease dementia or mild cognitive impairment [5]. The influence of the study partner type on the accuracy of information reporting has not been studied in preclinical AD.

Here, we describe analyses of data from the Alzheimer’s Disease Cooperative Study-Prevention Instrument (ADCS-PI) project. This project set out to instruct the design of AD prevention trials by enrolling and longitudinally following older cognitively unimpaired participants and their study partners in a design that simulates preclinical AD trials. Specifically, we investigated the integrity of participant and study partner reporting in this setting. Because the study partner requirement is a barrier to recruitment, we consider whether the study partner provides additional information beyond that provided by the participant.

## Methods

### Data source

The ADCS-PI design and participants have been described elsewhere [6]. Briefly, the protocol included participants 75 years of age or older who were fluent in English or Spanish. Participants were required to be in good general health and to be on stable doses of all medications for at least 4 weeks prior to screening. Participants were also required to be either cognitively normal or have possible MCI, although in our study we included only those who were cognitively normal (defined by a Clinical Dementia Rating Scale [CDR]-Global score of 0 at baseline) [7]. Participants had to be willing and able to participate in a 4- to 5-year study and be able to complete study procedures. They were also required to enroll with a study partner, with whom they had contact at least twice a week and who would be able to accompany them to all study visits. Participants were excluded if they had medical illnesses that would prevent their participation in the trial, alcohol or substance abuse within the past year, major psychiatric disorders, history of mental retardation, or were participating in a clinical drug trial. The study partner could not have a significant cognitive impairment, based on clinical impression or history known to the investigator, or be enrolled in the study [6].

Participants had annual visits for 4 years after enrollment into the study with a cognitive battery at each visit. Annual cognitive batteries included the modified Mini-Mental State Examination (mMMSE), Free and Cued Selective Reminding Test (FCSRT), New York University Paragraph Recall, Trail Making A and B, Wechsler Adult Intelligence Scale-Revised (WAIS-R) Digit Symbol Substitution Test, Boston Naming Test (10-item version), verbal fluency test (animals), and Geriatric Depression Scale (GDS). At the baseline visit, participants were randomized to take new experimental assessments either at home or in the clinic. All study participants provided written informed consent.

We used the participant and study partner versions of the Mail-In Cognitive Function Screening Instrument, which we refer to simply as the Cognitive Function Instrument (CFI), to compare the information provided by each member of the dyad. The CFI includes 2 sets of questions, 1 each for the participant and the study partner. Each set consists of 14 questions that ask about functional decline in the past year. The study partner and participant were asked to complete these questions independently, but the study partner was allowed to consult other people as long as it was not the participant. Questions were scored as “yes” (1 point), “no” (0 points), and “maybe” (0.5 points). The total score was calculated as the sum of the answers to each of the 14 questions, giving a possible range of 0 to 14. Several questions regarding driving, finances, and work performance had “not applicable” as another possible answer [8, 9]. For questions answered as “not applicable,” we imputed the mean of the available responses.

We used the modified Preclinical Alzheimer’s Cognitive Composite (mADCS-PACC) [9, 10] as an objective measure of cognition. This composite score was a combination of the original mADCS-PACC and the PACC-5 and included total recall from the Free and Cued Selective Reminding Test (FCSRT), New York University Paragraph Recall, Digit Symbol Substitution Test from the Wechsler Adult Intelligence Scale-Revised, mMMSE, and the animal fluency test [10, 11]. For each component, we calculated a *z*-score using the baseline sample mean and standard deviation for all participants with a CDR-Global score of 0. The *z*-scores across all components were averaged, and this value was used as the outcome, with lower scores indicating poorer cognition. If any components were missing, the outcome was calculated using the mean of the available components.

### Analyses

We used random forests to assess how well each member of the dyad predicted participants’ cognitive state at current and later visits. We used mADCS-PACC score as the response and included the baseline scores for CDR Sum of Boxes (CDR-SB) and participant and study partner CFI along with ethnicity, gender, age, education, and history of cardiovascular disease. Ten observations (0.7%) were missing 1 component of the mADCS-PACC, and 2 observations (0.2%) were missing 3 components. We used the GRF package in R to generate random forests. To account for variability stemming from the generation of the random forests, we created 100 random forests using the same data with different random seeds and obtained a variable importance measure each time. The variable importance measure used was a weighted sum of the number of times variables were selected for splitting, with splits higher in the tree having larger weight. We calculated the mean of the variable importance estimates, which we refer to as the estimated mean variable importance (eMVI), along with corresponding 95% uncertainty bounds (UB), which were obtained using the 2.5th and 97.5th percentiles of the 100 observed variable importance values. To investigate whether participant cognitive performance affected the observed relationships, we stratified participants using quartiles of the mADCS-PACC at baseline. The lowest quartile included participants with the poorest cognitive performance while the highest quartile included participants with the highest cognitive performance. To quantify differences in predictive performance between study partner types, we further stratified participants into spousal and non-spousal dyads. We also fit linear regression models for each stratum adjusting for the same variables that were used to generate the random forest results. To investigate how well current participant and study partner CFI scores predict current cognitive state, we repeated this procedure for each of the 4 years using cross-sectional scores for the mADCS-PACC, CFI, and CDR-SB.

Fifteen observations were excluded from the baseline analysis because either baseline participant or study partner CFI was missing. Seventy-two observations were excluded from the cross-sectional analysis because cross-sectional participant or study partner CFI was missing. Participants who dropped out were asked to return for a final visit. These visits were included in our analyses as the closest annual visit. Two treatment discontinuation visits were removed from our analysis because the participants already had a visit for the year closest to the treatment discontinuation. Three participants who were missing mADCS-PACC at baseline were also excluded from the cross-sectional analysis.

## Results

### Description of the sample

Our analyses included 450 participants, 189 who had a spousal study partner and 261 who had a non-spousal study partner (Table 1). Participants in the dyad groups were similar in age, education, mMMSE score, and CDR-SB score. The non-spousal group included more females (75.9% compared to 36.5%) and more non-Caucasians (30.3% compared to 8.5%) than did the spousal group. In both groups, participants rated their cognition worse than their partners did, though this difference was larger among non-spousal compared to spousal dyads. Study partners in the non-spousal group were younger and more often females, compared to the spousal group.

**Table 1:** Baseline description of ADCS-PI participants with a CD-Global score of 0 at baseline and at least 1 mADCS-PACC score and participant and study partner CFI available after baseline

	Spousal dyads (*N* = 189)	Non-spousal dyads (*N* = 261)
Participant characteristics		
Age, mean (SD)	78.92 (3.37)	79.77 (3.75)
Years of education, mean (SD)	15.59 (2.79)	14.80 (3.01)
Female sex, *n* (%)	69 (36.51)	198 (75.86)
Caucasian race, *n* (%)	173 (91.53)	182 (69.73)
mMMSE, mean (SD)	96.25 (2.97)	95.43 (3.75)
CDR-SB, mean (SD)	0.07 (0.18)	0.10 (0.20)
Participant CFI, mean (SD)	1.81 (1.88)	2.16 (2.04)
History of cardiovascular disease, *n* (%)	119 (62.96)	178 (68.20)
mADCS-PACC, mean (SD)	0.06 (0.62)	0.01 (0.70)
Study partner characteristics		
Study partner age, mean (SD)	76.08 (6.45)	64.08 (14.88)
Study partner female sex, *n* (%)	120 (63.49)	217 (83.14)
Study partner CFI, mean (SD)	1.10 (1.74)	1.04 (1.58)
Total contact time (days), mean (SD)	7.00 (0.00)	4.58 (2.04)

### Prospective prediction of future mADCS-PACC score

**Fig. 1:**
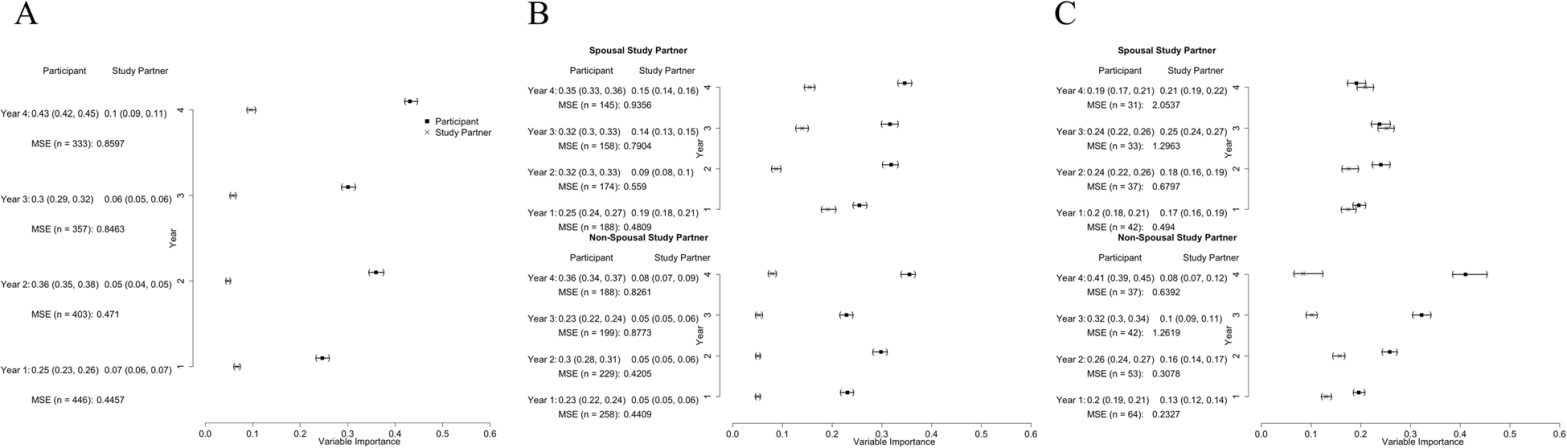
Forest plots presenting the estimated mean variable importance and 95% uncertainty bounds marginally (**a**), stratified by study partner type (**b**), and for the lowest (poorest performing) quartile of baseline mADCS-PACC (**c**) using baseline participant and study partner CFI

We first examined whether baseline CFI predicted mADCS-PACC scores at subsequent time points. We found that the eMVI for participant CFI was higher than that for study partner CFI (Fig. 1a). In the first year, the eMVI for participant CFI was 0.25 (95% UB: 0.23, 0.26) and that of the study partner was 0.07 (95% UB: 0.06, 0.07). This remained true when we stratified by study partner type (spousal vs. non-spousal), as seen in Fig. 1b. For example, the eMVI among spousal dyads for predicting cognitive performance after the first year was 0.25 (95% UB: 0.24, 0.27) for participant CFI and 0.19 (95% UB: 0.18, 0.21) for study partner CFI. Among non-spousal dyads, the eMVI was 0.23 (95% UB: 0.22, 0.24) and 0.05 (95% UB: 0.05, 0.06) for participant and study partner CFI, respectively. When we stratified by quartiles of cognitive performance (defined by mADCS-PACC scores at baseline), we found that among the poorest-performing participants with spousal study partners, the participant and study partner performed similarly (Fig. 1c). Among non-spousal dyads, the eMVI was higher for participant CFI all 4 years and the difference between participant and study partner CFI increased over time. In year 1, for example, the eMVI was 0.20 (95% UB: 0.19, 0.21) for participant CFI and 0.13 (95% UB: 0.12, 0.14) for study partner CFI. In year 4, the eMVI was 0.41 (95% UB: 0.39, 0.45) and 0.08 (95% UB: 0.07, 0.12) for participant and study partner CFI, respectively (Fig. 1c). The coefficient estimates also increased in magnitude over time. In the other quartiles, we found that the eMVI for participant CFI was usually higher than that of the study partner CFI (see Additional file 1).

### Cross-sectional prediction of current mADCS-PACC score

**Fig. 2:**
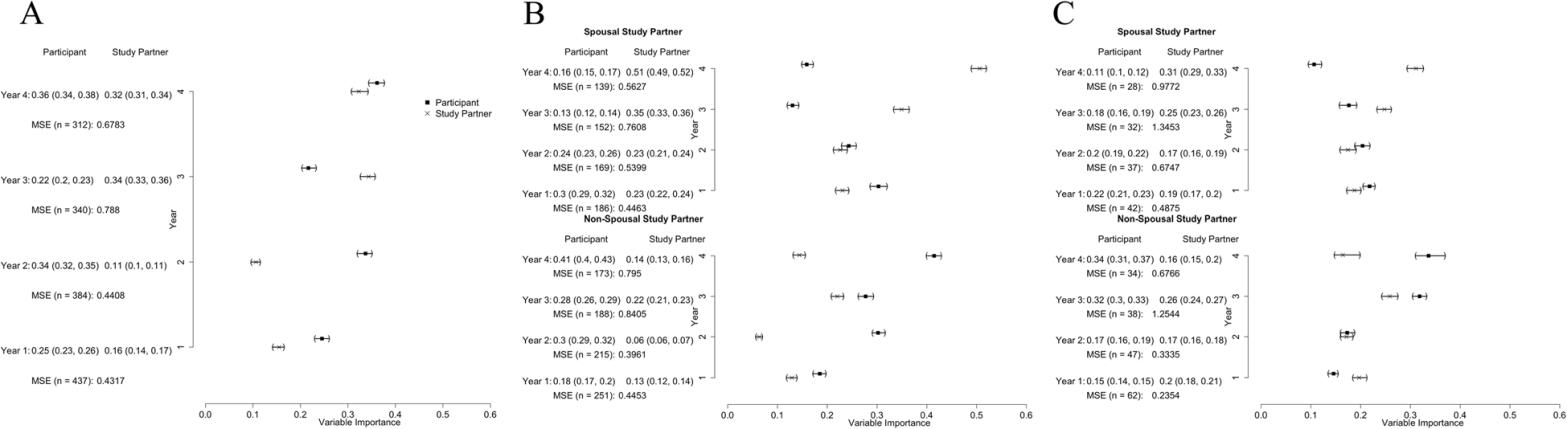
Forest plots presenting the estimated mean variable importance and 95% uncertainty bounds marginally (**a**), stratified by study partner type (**b**), and for the lowest (poorest performing) quartile of baseline mADCS-PACC (**c**) using cross-sectional participant and study partner CFI

We also examined the performance of participant and study partner CFI for predicting current mADCS-PACC scores. There were no clear trends between participant and study partner CFI. In years 1, 2, and 4, the eMVI was larger for the participant than for the study partner CFI while in year 3, the eMVI was higher for the study partner CFI (Fig. 2a). When we stratified by study partner type, we observed differences between the study partner and participant. As seen in Fig. 2b, among spousal dyads, the eMVI for participant CFI was higher than the study partner eMVI in the first 2 years; in years 3 and 4, the eMVI was higher for study partners. Among non-spousal dyads, the eMVI for participant CFI was higher than that for study partner CFI all 4 years, and the largest difference was observed in year 4 (Fig. 2b). In year 4, the participant CFI was 0.41 (95% UB: 0.40, 0.43) while that of the study partner, CFI was 0.14 (95% UB: 0.13, 0.16). Figure 2c illustrates the eMVIs for participants and study partners in the poorest-performing quartile over the course of the study, again defined based on the mADCS-PACC scores at baseline. As with the full sample, in spousal dyads, the participant performed better during the first 2 years while the study partner performed better during the last 2 years. Among non-spousal dyads, the study partner and participant performed similarly during the first 2 years. In years 3 and 4, the eMVI for participant CFI was higher than that for study partner CFI. The results for the other quartiles can be found in Additional file 1.

## Discussion

In this study, we investigated whether participant or study partner report was better at predicting cognitive performance in a study population intended to model AD prevention trial samples. We focused on individuals who were at greatest risk to show a cognitive decline—those with the lowest performance at baseline—since the source dataset did not include AD biomarkers, which are used to enrich preclinical AD trials and ensure statistical power [1]. We found that participants were better at predicting future cognitive performance (Fig. 1c) but that among spousal dyads, partners increasingly outperformed participants in predicting cognitive performance cross-sectionally over the course of study (Fig. 2c). This observation, which is similar to previous studies [12, 13], including previous analyses of this dataset [9, 14], suggests that knowledgeable informants may play an increasingly important role in identifying and quantifying cognitive changes over time in AD prevention trial participants.

This paper has several unique strengths. Our results are based on a large sample (*n* = 450), and we used a flexible prediction model to consider the participant and study partner performance. The model included study partner and participant CFI simultaneously, allowing us to compare the importance of each source of information. Our model also included other factors likely to predict CFI. By fitting linear models, we were also able to assess the magnitude and directionality of the relationships.

Another important strength of the current study is that we examined study partner characteristics as potential effect modifiers. When we stratified our analyses by dyad type, we found that among those with the poorest cognition, spousal study partners performed similarly to the participants in predicting mADCS-PACC scores, while participants with non-spousal study partners substantially outperformed their partners (Fig. 1c). When predicting the current cognitive state, we found no clear trends between the participant and study partner performance overall. But when we stratified our analyses by study partner type, we found that spousal study partners performed better than participants at later times. Participants without a spouse outperformed their partners in predicting cognitive performance, even at later time points (Fig. 2b, c).

Though these results are novel in relation to assessing study partner performance in AD prevention/preclinical AD trials, they are supported by observations in dementia and MCI. For example, Ready and colleagues showed that study partners who live with impaired participants (dementia and MCI) provide subjective reports that most strongly correlated with participant performance on a wordlist memory task, and these partners were most often spouses [5]. Non-spousal dyads are also at increased risk for dropout and partner replacement [15, 16], both of which can increase trial variance. Determining whether something specific to the nature of the relationship drives this effect or if it is some other factor such as residence status or the type, quantity, or quality of interactions between dyad members will require further research. Future research should also focus on methods to improve the integrity of the data provided by non-spousal partners, especially since they are underrepresented in trials and could expedite AD research if enrolled with greater frequency [17].

Our results indicate that all participants, regardless of their partner type, provide information about their future cognition that is at least as accurate as the information provided by partners. In most cases, participants better predict their future performance than do their partners. This observation may be important when considering optimal means to recruit to AD prevention trials, for example, from large registries. Depending on the goals and design of the specific trial, participant or partner reports of cognitive performance may be prioritized. It may also be important to the design of registries, since securing dyadic participation in registries necessitates added burden and cost. Nonetheless, having both reports, along with objective measures of cognitive performance, enables coordinated approaches that likely yield higher precision [18].

Overall, these results support the continued practice of dyadic enrollment in prevention/preclinical AD trials for the value partners bring to assessing cognitive performance. Study partners may also help participants deal with anxiety and distress related to AD risk disclosure and reduce study dropout by providing support networks for participants. Enrolling with a study partner may also allow the partner to become a trusted advocate who can facilitate planning and knows how the participant would like to be cared for in the case that they develop dementia [19]. Nevertheless, our results also indicate that careful consideration for the requirements of this role may be needed to optimize data integrity.

### Limitations

Several limitations of this study should be noted. Preclinical AD trials now enroll based on genetic and biomarker criteria as a strategy to identify participants at greatest risk for disease, to enrich for those most likely to benefit from therapy, and to improve statistical power [1, 20]. Although we did not have these variables to include in our analyses, we stratified by cognitive performance in an effort to replicate their purpose. We used a modified version of the mADCS-PACC to quantify cognition and CFI to assess participant and study partner views about the participant’s cognitive state. It is unclear whether our results are generalizable to other available tools, either for assessing participant cognitive performance [21] or subjective assessment of that performance [22]. In an accompanying paper by our group, however, we observed that these results generalize to the measurement of subjective assessments with the Everyday Cognition (ECog) scale and objective assessment with the Alzheimer’s Disease Assessment Scale-Cognitive-13 (ADAS-Cog-13) [23]. Finally, like actual interventional trials, the ADCS PI was subject to sample bias such as the inclusion of highly educated participants.

## Conclusions

Our analyses show that participants at risk for cognitive impairment with non-spousal study partners tend to be better at predicting their future cognitive performance than are their study partners. Spousal study partners, however, outperform participants in recognizing current cognitive performance at later time points. Therefore, study partner characteristics, in this study assessed based on the relationship between the partner and the participant, seem to modify the relationship between informant report and objective trial data. Thus, these results suggest that the study partner role may be important to the integrity of preclinical AD trial data but warrant further research to investigate ideal informant characteristics and interventions to increase the integrity of the data they provide.

## Additional file

**Additional file 1: Table S1.** Coefficient estimates for participant and study partner baseline CFI scores based on linear models in which the response is mADCS-PACC at each year and we adjust for each year’s scores of participant and study partner CFI, CDRSB, ethnicity, gender, age, education, and history of cardiovascular disease. **Table S2.** Coefficient estimates for participant and study partner CFI based on linear models in which the response is mADCS-PACC at each year and we adjust for participant and study partner CFI and CDRSB at each year along with ethnicity, gender, age, education, and history of cardiovascular disease. **Table S3.** Coefficient estimates corresponding to the linear models with baseline CFI. **Table S4.** Estimated mean variable importance (eMVI) and 95% UB for random forests with baseline CFI measures. **Table S5.** Coefficient estimates corresponding to the linear models with cross-sectional CFI. **Table S6.** Estimated mean variable importance (eMVI) and 95% UB for random forests with cross-sectional CFI measures.

## Data Availability

Data used in the preparation of this manuscript were obtained from the Alzheimer’s Disease Cooperative Study legacy database.

## Abbreviations

AD: Alzheimer’s disease
ADAS-Cog-13: Alzheimer’s Disease Assessment Scale-Cognitive-13
ADCS-PI: Alzheimer’s Disease Cooperative Study Prevention Instrument
ADL-PI: Activities of Daily Living-Prevention Instrument
CDR: Clinical Dementia Rating
CDR-SB: Clinical Dementia Rating Sum of Boxes
CFI: Cognitive Function Instrument
ECog: Everyday cognition
eMVI: Estimated mean variable importance
FCRST: Free and Cued Selective Reminding Test
GDS: Geriatric Depression Scale
mADCS-PACC: Modified Alzheimer’s Disease Cooperative Study-Preclinical Alzheimer’s Cognitive Composite
mMMSE: Modified Mini-Mental State Examination
QOL: Quality of life
UB: Uncertainty bounds
WAIS-R: Wechsler Adult Intelligence Scale-Revised
