# Supplementary material for: Study partner types and prediction of cognitive performance: implications to preclinical Alzheimer’s trials
Source: Alzheimers Res Ther. 2019 Nov 27;11:92. doi: 10.1186/s13195-019-0544-6 (PMC6881999; doi:10.1186/s13195-019-0544-6)
Supplement: Supplementary file 1 — Additional file 1: Table S1. Coefficient estimates for participant and study partner baseline CFI scores based on linear models in which the response is mADCS-PACC at each year and we adjust for each year’s scores of participant and study partner CFI, CDRSB, ethnicity, gender, age, education, and history of cardiovascular disease. Table S2. Coefficient estimates for participant and study partner CFI based on linear models in which the response is mADCS-PACC at each year and we adjust for participant and study partner CFI and CDRSB at each year along with ethnicity, gender, age, education, and history of cardiovascular disease. Table S3. Coefficient estimates corresponding to the linear models with baseline CFI. Table S4. Estimated mean variable importance (eMVI) and 95% UB for random forests with baseline CFI measures. Table S5. Coefficient estimates corresponding to the linear models with cross-sectional CFI. Table S6. Estimated mean variable importance (eMVI) and 95% UB for random forests with cross-sectional CFI measures. [file 13195_2019_544_MOESM1_ESM.docx]

|  | **Spousal** | | **Non-Spousal** | | **Combined** | |
| --- | --- | --- | --- | --- | --- | --- |
|  | **Participant** | **Study Partner** | **Participant** | **Study Partner** | **Participant** | **Study Partner** |
| **Overall (All Quartiles)** | | | | | | |
| **Year 1** | -0.035  (-0.093, 0.022) | -0.048  (-0.111, 0.016) | -0.070  (-0.111, -0.030) | -0.030  (-0.081, 0.021) | -0.061  (-0.094, -0.028) | -0.032  (-0.072, 0.007) |
| **Year 2** | -0.072  (-0.138, -0.007) | 0.016  (-0.061, 0.093) | -0.089  (-0.131, -0.047) | -0.015  (-0.066, 0.036) | -0.086  (-0.121, -0.050) | 0.000  (-0.043, 0.043) |
| **Year 3** | -0.103  (-0.186, -0.020) | 0.004  (-0.094, 0.102) | -0.085  (-0.151, -0.019) | -0.044  (-0.125, 0.037) | -0.100  (-0.151, -0.049) | -0.022  (-0.084, 0.040) |
| **Year 4** | -0.198  (-0.299, -0.098) | -0.025  (-0.140, 0.090) | -0.117  (-0.187, -0.047) | -0.042  (-0.125, 0.040) | -0.149  (-0.206, -0.092) | -0.037  (-0.104, 0.029) |
| **First Quartile (Poorest Cognitive Performance)** | | | | | | |
| **Year 1** | -0.012  (-0.109, 0.085) | -0.012  (-0.113, 0.088) | -0.023  (-0.070, 0.025) | -0.064  (-0.128, -0.001) | -0.028  (-0.073, 0.018) | -0.036  (-0.089, 0.017) |
| **Year 2** | -0.015  (-0.145, 0.115) | 0.031  (-0.115, 0.177) | -0.053  (-0.114, 0.007) | -0.059  (-0.135, 0.016) | -0.042  (-0.102, 0.017) | -0.013  (-0.083, 0.058) |
| **Year 3** | -0.060  (-0.256, 0.135) | -0.006  (-0.220, 0.208) | -0.140  (-0.280, 0.001) | -0.070  (-0.250, 0.109) | -0.111  (-0.223, 0.001) | -0.025  (-0.159, 0.109) |
| **Year 4** | -0.171  (-0.420, 0.078) | 0.033  (-0.240, 0.305) | -0.175  (-0.288, -0.063) | -0.006  (-0.134, 0.123) | -0.183  (-0.299, 0.067) | 0.018  (-0.116, 0.151) |
| **Second Quartile** | | | | | | |
| **Year 1** | 0.004  (-0.112, 0.120) | -0.126  (-0.304, 0.052) | -0.063  (-0.134, 0.007) | -0.035  (-0.121, 0.052) | -0.054  (-0.112, 0.004) | -0.035  (-0.112, 0.042) |
| **Year 2** | -0.087  (-0.219, 0.044) | 0.137  (-0.084, 0.358) | -0.048  (-0.116, 0.020) | 0.009  (-0.073, 0.091) | -0.062  (-0.118, -0.006) | 0.035  (-0.040, 0.110) |
| **Year 3** | -0.093  (-0.256, 0.070) | 0.144  (-0.172, 0.460) | -0.009  (-0.112, 0.093) | -0.071  (-0.188, 0.047) | -0.018  (-0.096, 0.061) | -0.041  (-0.142, 0.061) |
| **Year 4** | -0.205  (-0.359, -0.051) | 0.270  (0.006, 0.534) | -0.092  (-0.252, 0.067) | -0.093  (-0.274, 0.087) | -0.098  (-0.201, 0.006) | -0.071  (-0.205, 0.064) |
| **Third Quartile** | | | | | | |
| **Year 1** | -0.081  (-0.176, 0.015) | 0.103  (-0.008, 0.213) | -0.012  (-0.069, 0.044) | -0.015  (-0.077, 0.048) | -0.022  (-0.069, 0.025) | 0.029  (-0.023, 0.080) |
| **Year 2** | -0.038  (-0.140, 0.063) | 0.066  (-0.054, 0.185) | -0.018  (-0.082, 0.046) | 0.042  (-0.026, 0.109) | -0.035  (-0.087, 0.018) | 0.039  (-0.016, 0.094) |
| **Year 3** | -0.032  (-0.195, 0.131) | 0.111  (-0.055, 0.277) | -0.009  (-0.161, 0.142) | 0.058  (-0.118, 0.235) | -0.041  (-0.147, 0.065) | 0.051  (-0.065, 0.166) |
| **Year 4** | -0.092  (-0.246, 0.063) | 0.060  (-0.121, 0.240) | 0.107  (-0.045, 0.259) | 0.042  (-0.109, 0.193) | 0.001  (-0.096, 0.099) | 0.052  (-0.052, 0.155) |
| **Fourth Quartile (Best Cognitive Performance)** | | | | | | |
| **Year 1** | 0.068  (-0.039, 0.176) | -0.135  (-0.253, -0.016) | -0.045  (-0.118, 0.029) | 0.140  (0.041, 0.239) | 0.003  (-0.061, 0.066) | -0.011  (-0.087, 0.066) |
| **Year 2** | -0.015  (-0.114, 0.085) | -0.049  (-0.161, 0.063) | -0.121  (-0.203, -0.040) | 0.092  (-0.012, 0.195) | -0.052  (-0.114, 0.010) | 0.025  (-0.049, 0.099) |
| **Year 3** | -0.061  (-0.160, 0.038) | -0.018  (-0.130, 0.093) | -0.105  (-0.196, -0.014) | 0.062  (-0.057, 0.181) | -0.065  (-0.129, -0.001) | 0.011  (-0.067, 0.089) |
| **Year 4** | -0.045  (-0.255, 0.166) | -0.109  (-0.296, 0.079) | -0.064  (-0.170, 0.042) | 0.017  (-0.116, 0.150) | -0.045  (-0.141, 0.050) | -0.052  (-0.157, 0.053) |

*Table S1: Coefficient estimates for participant and study partner baseline CFI scores based on linear models in which the response is mADCS-PACC at each year and we adjust for each year’s scores of participant and study partner CFI, CDRSB, ethnicity, gender, age, education, and history of cardiovascular disease.*

|  | **Spousal** | | **Non-Spousal** | | **Combined** | |
| --- | --- | --- | --- | --- | --- | --- |
|  | **Participant** | **Study Partner** | **Participant** | **Study Partner** | **Participant** | **Study Partner** |
| **Overall (All Quartiles)** | | | | | | |
| **Year 1** | -0.051  (-0.105, 0.002) | -0.075  (-0.133,-0.017) | -0.045  (-0.082,-0.008) | -0.049  (-0.083, -0.016) | -0.051  (-0.081,-0.021) | -0.052  (-0.081, -0.023) |
| **Year 2** | -0.054  (-0.110, 0.003) | -0.053  (-0.107, 0.000) | -0.073  (-0.112,-0.034) | -0.026  (-0.059, 0.007) | -0.062  (-0.094,-0.030) | -0.033  (-0.062, -0.004) |
| **Year 3** | -0.002  (-0.070, 0.066) | -0.126  (-0.189,-0.063) | -0.105  (-0.163,-0.047) | -0.052  (-0.094,-0.011) | -0.070  (-0.113,-0.026) | -0.071  (-0.105,-0.037) |
| **Year 4** | -0.056  (-0.118, 0.006) | -0.136  (-0.184,-0.088) | -0.144  (-0.196,-0.092) | -0.020  (-0.065, 0.024) | -0.107  (-0.146,-0.068) | -0.061  (-0.094,-0.028) |
| **First Quartile (Poorest Cognitive Performance)** | | | | | | |
| **Year 1** | 0.027  (-0.071, 0.124) | -0.083  (-0.190, 0.023) | -0.010  (-0.055, 0.034) | -0.025  (-0.064, 0.014) | -0.001  (-0.045, 0.043) | -0.034  (-0.074, 0.006) |
| **Year 2** | 0.011  (-0.094, 0.116) | -0.036  (-0.139, 0.067) | -0.009  (-0.068, 0.051) | -0.040  (-0.099, 0.019) | -0.002  (-0.056, 0.052) | -0.033  (-0.085, 0.019) |
| **Year 3** | -0.006  (-0.196, 0.184) | -0.027  (-0.192,0.138) | -0.074  (-0.198, 0.051) | -0.075  (-0.175, 0.025) | -0.053  (-0.154, 0.048) | -0.082  (-0.159, -0.004) |
| **Year 4** | -0.033  (-0.181, 0.115) | -0.128  (-0.243, -0.013) | -0.117  (-0.211,-0.022) | -0.016  (-0.100, 0.067) | -0.079  (-0.157, -0.001) | -0.070  (-0.135, -0.006) |
| **Second Quartile** | | | | | | |
| **Year 1** | -0.037  (-0.116, 0.042) | -0.042  (-0.190, 0.106) | -0.052  (-0.109, 0.005) | -0.027  (-0.079, 0.025) | -0.053  (-0.099, -0.007) | -0.033  (-0.080, 0.014) |
| **Year 2** | -0.051  (-0.128, 0.025) | -0.051  (-0.176, 0.073) | -0.029  (-0.087, 0.028) | 0.017  (-0.028, 0.063) | -0.045  (-0.090, 0.000) | 0.015  (-0.028, 0.058) |
| **Year 3** | -0.115  (-0.205, -0.026) | -0.030  (-0.134, 0.074) | -0.089  (-0.185, 0.007) | -0.024  (-0.074, 0.025) | -0.093  (-0.157, -0.029) | -0.027  (-0.066, 0.013) |
| **Year 4** | -0.064  (-0.139, 0.011) | -0.035  (-0.099, 0.029) | -0.204  (-0.311,-0.098) | 0.044  (-0.078, 0.166) | -0.160  (-0.227,-0.093) | 0.006  (-0.061, 0.072) |
| **Third Quartile** | | | | | | |
| **Year 1** | -0.014  (-0.127, 0.099) | -0.044  (-0.143, 0.055) | -0.015  (-0.065, 0.035) | -0.022  (-0.077, 0.034) | -0.015  (-0.060, 0.031) | -0.021  (-0.067, 0.025) |
| **Year 2** | 0.047  (-0.065, 0.160) | -0.070  (-0.135,-0.004) | -0.060  (-0.143, 0.023) | 0.040  (-0.013, 0.093) | -0.026  (-0.089, 0.037) | -0.008  (-0.049, 0.032) |
| **Year 3** | 0.093  (-0.040, 0.227) | -0.251  (-0.389,-0.113) | -0.127  (-0.276, 0.022) | 0.026  (-0.080, 0.132) | -0.055  (-0.164, 0.053) | -0.023  (-0.107, 0.060) |
| **Year 4** | -0.007  (-0.155, 0.141) | -0.187  (-0.327,-0.047) | -0.005  (-0.152, 0.142) | 0.028  (-0.066, 0.121) | -0.013  (-0.115, 0.089) | -0.011  (-0.082, 0.059) |
| **Fourth Quartile (Best Cognitive Performance)** | | | | | | |
| **Year 1** | -0.103  (-0.216, 0.010) | -0.028  (-0.154, 0.099) | -0.025  (-0.102, 0.051) | -0.010  (-0.102, 0.083) | -0.056  (-0.115, 0.003) | -0.028  (-0.099, 0.043) |
| **Year 2** | 0.005  (-0.119, 0.128) | -0.058  (-0.165, 0.050) | -0.104  (-0.176,-0.032) | -0.025  (-0.089, 0.038) | -0.048  (-0.105, 0.010) | -0.029  (-0.082, 0.024) |
| **Year 3** | 0.085  (-0.002, 0.172) | -0.165  (-0.261,-0.069) | -0.009  (-0.082, 0.065) | -0.008  (-0.069, 0.053) | 0.008  (-0.042, 0.058) | -0.062  (-0.111, -0.013) |
| **Year 4** | -0.003  (-0.144, 0.138) | -0.148  (-0.254,-0.041) | -0.049  (-0.115, 0.018) | -0.044  (-0.092, 0.004) | -0.031  (-0.091, 0.029) | -0.079  (-0.125, -0.033) |

*Table S2: Coefficient estimates for participant and study partner CFI based on linear models in which the response is mADCS-PACC at each year and we adjust for participant and study partner CFI and CDRSB at each year along with ethnicity, gender, age, education, and history of cardiovascular disease.*


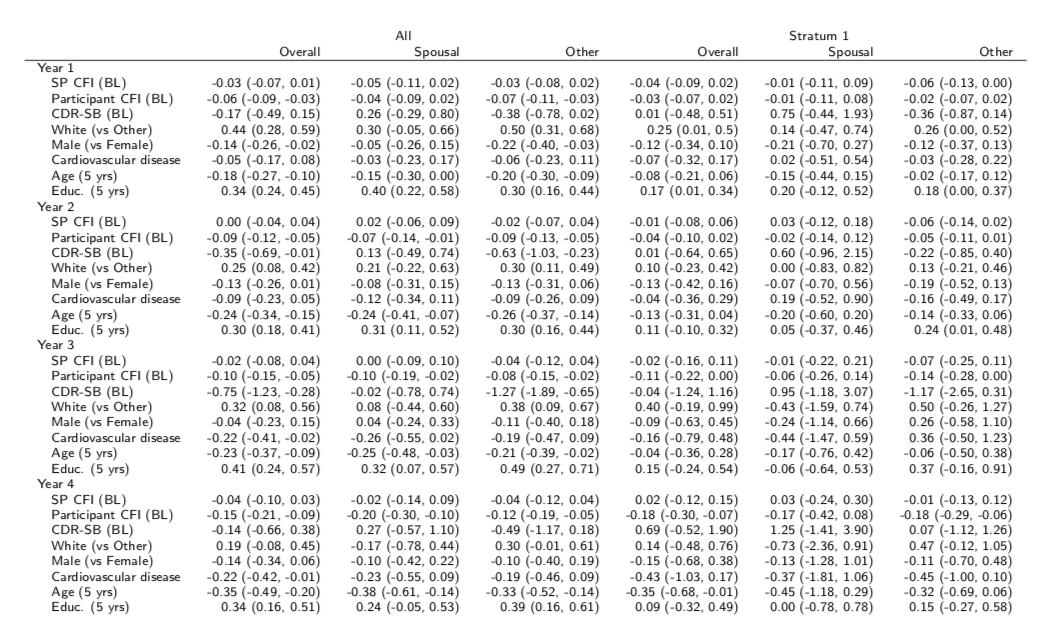


*Table S3: Coefficient estimates corresponding to the linear models with baseline CFI.*

*
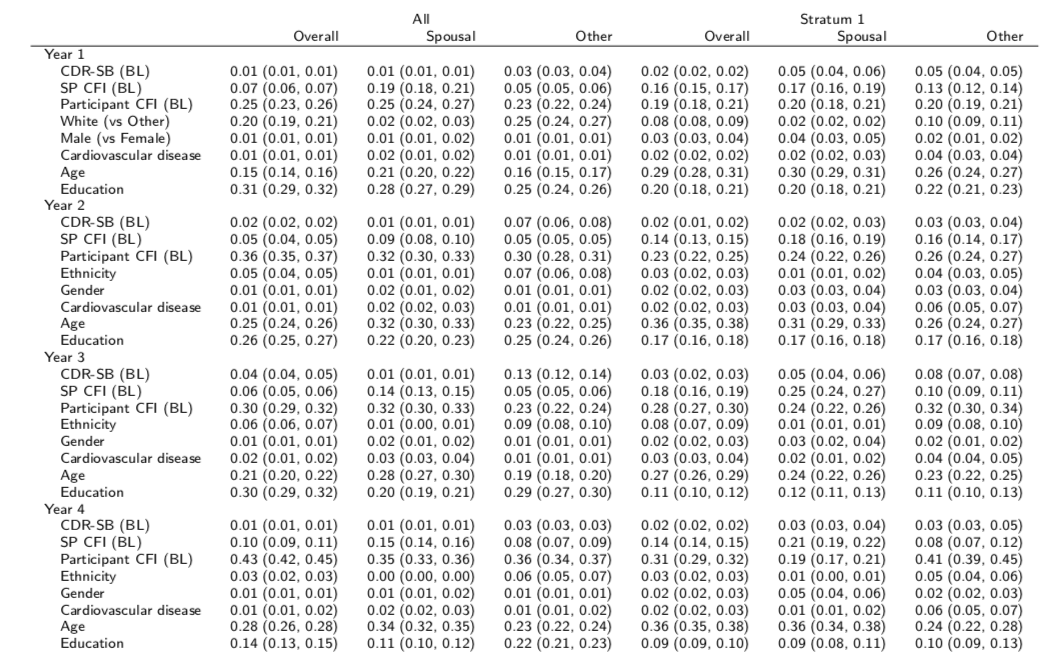
*

*Table S4: Estimated mean variable importance (eMVI) and 95% UB for random forests with baseline CFI measures.*

*
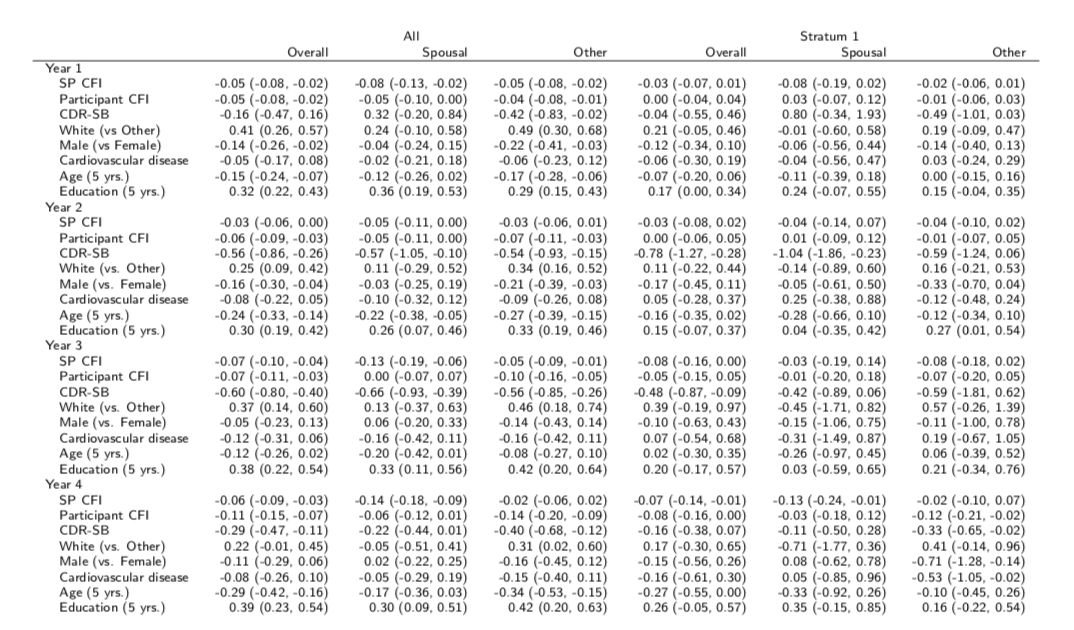
*

*Table S5: Coefficient estimates corresponding to the linear models with cross-sectional CFI.*

*
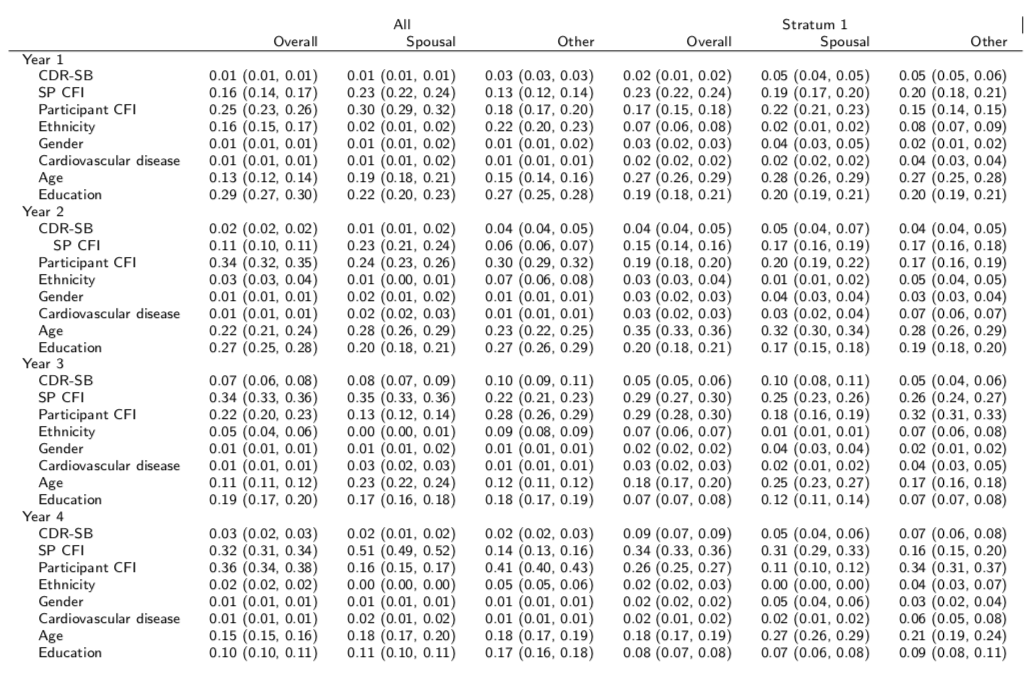
*

*Table S6: Estimated mean variable importance (eMVI) and 95% UB for random forests with cross-sectional CFI measures.*
